# Patient Experience with Intranasal Esketamine in Treatment-Resistant Depression: Insights from a Multicentric Italian Study (REAL-ESKperience)

**DOI:** 10.3390/jpm15040161

**Published:** 2025-04-21

**Authors:** Marco Di Nicola, Maria Pepe, Giacomo d’Andrea, Ilaria Marcelli, Mauro Pettorruso, Ileana Andriola, Stefano Barlati, Matteo Carminati, Carlo Ignazio Cattaneo, Massimo Clerici, Domenico De Berardis, Sergio De Filippis, Bernardo Dell’Osso, Giorgio Di Lorenzo, Giuseppe Maina, Mirko Manchia, Matteo Marcatili, Vassilis Martiadis, Cinzia Niolu, Antonino Petralia, Gianluca Rosso, Gianluca Serafini, Maria Salvina Signorelli, Tommaso Vannucchi, Matteo Vismara, Raffaella Zanardi, Antonio Vita, Gabriele Sani, Giovanni Martinotti, REAL-ESKperience Study Group

**Affiliations:** 1 Department of Neuroscience, Section of Psychiatry, Università Cattolica del Sacro Cuore, 00168 Roma, Italy; 2 Department of Psychiatry, Fondazione Policlinico Universitario Agostino Gemelli IRCCS, 00168 Roma, Italy; 3 Department of Neurosciences, Imaging and Clinical Sciences, Università degli Studi G. d’Annunzio, 66100 Chieti, Italy; 4 Department of Translational Biomedicine and Neuroscience, Università degli Studi di Bari Aldo Moro, 70121 Bari, Italy; 5 Department of Clinical and Experimental Sciences, University of Brescia, 25123 Brescia, Italy; 6 Department of Mental Health and Addiction Services, ASST Spedali Civili of Brescia, 25123 Brescia, Italy; 7 Mood Disorder Unit, Department of Clinical Neurosciences, IRCCS San Raffaele Scientific Institute, 20132 Milan, Italy; 8 Department of Mental Health, ASL Biella, 13875 Biella, Italy; 9 Department of Mental Health, Fondazione IRCCS San Gerardo dei Tintori, 20900 Monza, Italy; 10 School of Medicine and Surgery, University of Milano Bicocca, 20126 Milan, Italy; 11 Department of Mental Health, ASL 4, 64100 Teramo, Italy; 12 Neuropsychiatric Clinic, Villa Von Siebenthal, 00040 Genzano di Roma, Italy; 13 Department of Biomedical and Clinical Sciences Luigi Sacco and Aldo Ravelli Center for Neurotechnology and Brain Therapeutic, Università di Milano, 20132 Milano, Italy; 14 Chair of Psychiatry, Department of Systems Medicine, Tor Vergata, 00133 Roma, Italy; 15 IRCCS Fondazione Santa Lucia, 00179 Roma, Italy; 16 San Luigi Gonzaga University Hospital, Department of Neurosciences “Rita Levi Montalcini”, Università di Torino, 10125 Torino, Italy; 17 Unit of Clinical Psychiatry, University Hospital Agency of Cagliari, 09123 Cagliari, Italy; 18 Department of Pharmacology, Dalhousie University, Halifax, NS B3H 2Y9, Canada; 19 Department of Mental Health, ASL Napoli 1 Centro, 80145 Naples, Italy; 20 Department of Clinical and Experimental Medicine, Psychiatry Unit, Università di Catania, 95123 Catania, Italy; 21 Department of Neuroscience, Rehabilitation, Ophthalmology, Genetics, Maternal and Child Health (DINOGMI), Section of Psychiatry, University of Genova, 17100 Genova, Italy; 22 IRCCS Ospedale Policlinico San Martino, 16132 Genova, Italy; 23 Functional Unit of Adults Mental Health, Mental Health Department, 59100 Prato, Italy; 24 Department of Clinical Neurosciences, Università Vita-Salute San Raffaele, 20132 Milano, Italy

**Keywords:** artificial intelligence, glutamatergic system, mood disorders, patient perspective, personalized medicine, psychopharmacology

## Abstract

**Background**. Treatment-resistant depression (TRD) is a prevalent, high-burden disorder. Esketamine nasal spray (ESK-NS) has been approved for, T.R.D.; and efficacy has been observed in both clinical trials and real-world studies. However, observations integrating patients’ perspective on this treatment are limited. This multicentric Italian study explored experiences with ESK-NS in TRD patients, focusing on perceived therapeutic effects and overall satisfaction. **Methods**. A self-report survey was administered to 236 outpatients with TRD (55.1% females, 54.1 ± 14.1 years) treated with ESK-NS for at least three consecutive months within standard clinical care. Based on satisfaction levels, participants were classified as “unsatisfied” (10.2%), “partially satisfied” (19.1%), “satisfied” (44.4%), or “very satisfied” (26.3%), and compared for sociodemographic, clinical characteristics, and feedback on perceived benefits. Artificial intelligence (OpenAI) served to categorize responses to an open-ended question. **Results**. Enhanced quality of life was reported by 88.4% of participants. Significant differences emerged in earliest self-perceived benefits, most relevant effects, and impact on global functioning across groups. Specifically, “very satisfied” patients described the following: early improvements in depressed mood, suicidal thoughts, and restlessness; decreased suicidal thoughts among the most significant effects; and functional gains across all domains. OpenAI identified experiences of personal growth and rediscovery and a desire for tailored settings and approaches as recurring topics. **Conclusions**. Most patients reported a positive perception of ESK-NS treatment. The most satisfied participants highlighted significant benefits to depressed mood, suicidal thoughts, and overall functioning. Patient-reported experiences offer insights into different psychopathological dimensions, including functional outcomes and quality of life. Integrating these perspectives into clinical practice might assist treatment personalization, improving patients’ adherence and satisfaction.

## 1. Introduction

Patient-centered approaches in psychiatric care are crucial for providing insight into the perceived helpfulness of treatments and their impact not only on symptom reduction but also on quality of life and functional outcomes [1]. Patients’ perspective might help to tailor interventions and better understand therapeutic effects, which are typically assessed through clinician-based objective measures in routine practice [2]. Similar approaches might be useful in challenging conditions where effective treatments are limited, like treatment-resistant depression (TRD), commonly defined as the failure to respond to at least two antidepressants, adequately assumed as for time, dosage, and adherence (i.e., the patient’s active and consistent engagement with the prescribed treatment) [3].

TRD is a significant public health concern because of its prevalence, with rates varying from 12% to 55%, and socioeconomic impact [4,5,6]. It can be detected in both major and bipolar depression [7] and frequently co-occurs with physical and other mental health diseases, leading to substantial functional impairments and reduced quality of life [8]. The intranasal formulation of esketamine, the S-enantiomer of ketamine, which acts as a noncompetitive N-methyl-D-aspartate receptor (NMDA-R) antagonist with great affinity, has been specifically approved for treating TRD and marks a pivotal shift from traditional monoaminergic approaches [9,10]. Several randomized trials and real-world studies have reported the effectiveness of esketamine nasal spray (ESK-NS) in combination with serotonergic drugs as well as its good tolerability, with transient side effects—most commonly dissociative symptoms, dizziness, nausea, and increased blood pressure—that typically peak within the first hour post-administration and resolve spontaneously [11,12,13,14]. Preliminary evidence is also available on its efficacy when administered with antidepressants with different mechanisms of action [15]. The approved treatment schedule includes twice-weekly administration for four weeks, followed by once-weekly dosing for another four weeks, and subsequently weekly or biweekly maintenance with a target dose of either 56 or 84 mg based on clinical response and tolerability [16].

Similarly to other pharmacological treatments, the effects of ESK-NS have been objectively evaluated by clinicians, while patients’ perspectives and experience have been assessed mainly through audio interviews and self-administered questionnaires that investigate either the impact on psychopathological dimensions (e.g., anhedonia, cognition, physical symptoms, and suicidality) [17,18,19] or on quality of life and functioning [20,21]. However, it has been described that patients undergoing treatment with esketamine report a range of phenomenological experiences that often extend beyond symptom reduction and may significantly impact therapeutic outcomes [22,23]. Integrating patient insights about the effect on psychopathology, functioning, and quality of life into ESK-NS protocols may lead to a deeper understanding of the wide range of implications of this treatment [24]. Indeed, patient-reported outcomes have been increasingly recognized as a major contribution to evaluating therapeutic success, as they can capture experiential factors that might guide clinicians in optimizing treatment adherence, satisfaction, and overall effectiveness [2,25].

To the best of our knowledge, this is the first study to employ a multicentric approach to examine, through the administration of a dedicated, self-report survey, the experiences of patients with TRD treated with ESK-NS across various Italian clinical settings. By focusing on the subjective experience of treatment, this research aims to describe the multidimensional impact of ESK-NS on patients’ mental health, daily lives, and overall well-being, possibly contributing to a thorough understanding of TRD in clinical practice.

## 2. Methods

### 2.1. Participants

This study was conducted through the administration of a survey to patients with a depressive episode that met the definition for TRD (i.e., insufficient response to two or more antidepressant treatments) who were treated with ESK-NS across several psychiatric units in Italy as part of the observational, multicentric REAL-ESK study [13]. In accordance with the national regulatory framework, ESK-NS was provided free of charge to all participants through the Italian National Health Service as part of routine clinical care in authorized public facilities, including community mental health centers and hospital-based services. Efforts were made to recruit patients from mental health facilities across different regions to ensure a sample as representative as possible of the Italian population. The participating psychiatric units included community-based centers, hospital-based or university-affiliated departments, and rehabilitative clinics from the public mental health system, reflecting the variability of service provision across Italian regions. Specifically, centers were involved from (a) Northern Italy (ASL Biella; University of Brescia; University of Genova; Milano Bicocca University; University of Milan; IRCCS San Raffaele Scientific Institute of Milan; Fondazione IRCCS San Gerardo dei Tintori, Monza; and San Luigi Gonzaga University Hospital of Torino); (b) Central Italy (G. d’Annunzio University of Chieti; Fondazione Policlinico Universitario A. Gemelli IRCCS of Rome; ALS 4 Teramo; ASL Toscana Centro UFSMA Prato; University of Rome Tor Vergata; and Von Siebenthal Clinic of Rome, Italy); and (c) Southern Italy (A. Moro University of Bari; University of Cagliari; University of Catania; and ASL Napoli 1).

Eligibility for this study was established according to the following criteria: age over 18 years; fluency in both written and spoken Italian; diagnosis of a current depressive episode falling under definition of TRD; and treatment with a target dose of ESK-NS in an outpatient setting for at least three consecutive months, administered in combination with selective serotonin reuptake inhibitors (SSRIs) and/or serotonin/norepinephrine reuptake inhibitors (SNRIs) [26]. The survey included both patients who had completed ESK-NS and those still in treatment at the time of data collection, provided they had been receiving it for at least three consecutive months to ensure sample homogeneity, and in line with the treatment schedule approved by national regulatory authorities (i.e., twice-weekly for four weeks, once-weekly for other four weeks, and then once-weekly or biweekly based on clinical judgment). This timeframe was also established in accordance with the greater response to ESK-NS observed at three months in real-world studies [13]. Patients were excluded if they were non-Italian speakers or if they were unwilling or unable to complete the questionnaire, due to cognitive deficits or other clinical impairments that would affect self-report reliability.

### 2.2. Procedure and Data Collection

A self-report questionnaire was set up to assess demographic and epidemiological variables of interest (i.e., age, gender, education level, occupation, marital status, and presence of a family/social support network). Clinical information such as the overall duration of treatment with ESK-NS and time since its interruption (when applicable), as well as the availability of concomitant psychoeducational support or psychotherapy, were collected. Specific questions were developed to investigate the effects of treatment as perceived by the patients, their overall rating about the experience with ESK-NS, and their suggestions for possible changes or improvements. Questions were designed to detect the effects of the compound on depressive symptomatic domains as indicated in DSM-5-TR [27] and to evaluate changes related to general functioning and quality of life.

The survey was administered during routine follow-up visits at the various psychiatric services, where patients were regularly followed as part of their ongoing treatment. It was provided by clinicians but completed independently by patients on site, either in paper or digital format depending on the technical resources available at each center. It consisted of 20 questions, including both single- and multiple-choice items, as well as a final open-ended question that allowed patients to describe the treatment experience in their own words. Completion time averaged approximately 10–15 min, based on preliminary piloting conducted to test clarity and usability. Although all items were technically mandatory, participants were informed that survey participation was entirely voluntary, and they could withdraw at any time. Confidentiality was ensured by anonymizing questionnaires at the time of completion, followed by further de-identification through data aggregation before analysis, in full adherence to European data protection regulations. The survey with all items is reported in Supplementary Material S1.

### 2.3. Data Analysis

The sample was subdivided into four groups according to satisfaction level based on question #18 of the survey: “unsatisfied”, “partially satisfied”, “satisfied”, and “very satisfied”. Descriptive data were summarized as the number of patients and percentage (%) for categorical variables or mean ± standard deviation (M ± SD) for continuous variables. Since all survey responses (except for ordinal ones) were dichotomized into binary-coded variables (0 = no, 1 = yes) during data processing, the numbers and percentages reported in the Results section represent the proportion of participants whose responses could be categorized as “yes” for questions where this recoding was applicable.

Comparisons among the four satisfaction groups were performed using ANOVA for continuous variables, whereas contingency tables, the Chi-square test, or Fisher’s Exact Test (when expected frequencies were <5) were used for dichotomous variables. For all significant associations identified with Chi-square tests, Cramer’s V coefficients were calculated to estimate the strength of the association. Effect sizes were interpreted as small (≤0.2), moderate (0.2–0.6), or strong (>0.6) [28]. Post hoc pairwise comparisons were conducted for significant between-group differences and Bonferroni correction was applied to adjust for multiple comparisons and minimize the likelihood of Type I errors, setting the corrected significance threshold at *α*_corrected_. The Chi-square test was used for post hoc analyses as well, except when expected frequencies were too low, where Fisher’s Exact Test was performed. Results of post hoc comparisons were visualized using a heatmap representing the magnitude of differences across groups. All analyses were conducted using IBM SPSS Statistics for Windows, v. 28 (IBM Corp., Armonk, NY, USA).

### 2.4. Thematic Categorization of Open-Ended Responses

A preliminary thematic clustering of open-field responses was conducted by the research team based on a full reading of all patient-provided texts. Consequently, artificial intelligence (AI) was used to extrapolate recurring topics and their relative percentages to support the refinement of thematic organization. Specifically, the qualitative analysis was conducted using ChatGPT-4 (November 2024 version) via OpenAI’s web interface [29]. Two predefined prompts were used to analyze the data: (1) “analyze the recurring themes in these open-ended responses and summarize them in key categories”; (2) “group these responses into thematic clusters and provide a percentage distribution of each theme”. The AI-generated outputs were reviewed and compared to the initial manual categorization to ensure consistency and accuracy. Themes were verified against the raw textual data and any inconsistencies or ambiguous classifications were resolved by consensus among human raters. Only fully anonymized textual excerpts were processed, with all potentially identifying information removed prior to submission. ChatGPT was accessed with customized privacy settings (i.e., chat history and data storage disabled), ensuring that no input was saved, retained, or used for model training. No statistical comparisons were performed across thematic categories, as this phase aimed to qualitatively summarize the feedback derived from patients’ open-ended responses. The resulting thematic classification was then visualized graphically to facilitate interpretation.

## 3. Results

A final sample of 236 patients from Northern (24.5%), Central (45.8%), and Southern (29.7%) Italy that fulfilled all inclusion criteria was included. Participants were mainly females (55.1%), unemployed (52.3%), in a current relationship (50%), had completed at least secondary education (high school or higher) (71.9%), and almost all reported to have a valid family/social support network (81.8%). More than half of the sample was receiving ESK-NS (56.6%) at the time of administration of the survey, had been in treatment for more than six months (54.9%), and was undergoing concomitant psychological therapy (52.3%).

Based on satisfaction level with treatment, patients were divided into “unsatisfied” (10.2%), “partially satisfied” (19.1%), “satisfied” (44.4%), and “very satisfied” (26.3%) groups. No significant sociodemographic and clinical differences were detected except for the presence of a valid family/social support network (*p* = 0.008, Cramer’s V: 0.236) and the time interval between discontinuation of ESK-NS treatment and administration of the survey (*p* = 0.007, Cramer’s V: 0.207). Sociodemographic and clinical characteristics of the overall sample and of subgroups are summarized in Table 1.

Overall, beneficial effects within the first month of treatment were reported in 40.3% of cases. Patients rated the improvement in depressed mood (74%) and the reduction in suicidal thoughts (36.6%), fatigue (33.9%), and anhedonia (33.5%) as the positive effects perceived earlier, describing the decrease in depressed mood (50.5%) and suicidal thoughts (16.7%) as the most significant. Overall, 89.8% of participants declared some degree of satisfaction with treatment. A positive impact on quality of life was reported in 88.4% of cases, with improvements in levels of affective–relational (50.7%) and social (47.6%) functioning. Sedation (39.6%) and dizziness (39.5%) were both described as the most uncomfortable effects during the observation period after the administration, followed by transient dissociative experiences (25.3%), while almost a third of the sample (30.2%) reported the absence of uncomfortable side effects.

Significant between-group differences emerged in the improvement in depressed mood (*p* = 0.014, Cramer’s V: 0.331), reduction in suicidal thoughts (*p* < 0.001, Cramer’s V: 0.351), restlessness (*p* = 0.003, Cramer’s V: 0.251), and feelings of guilt (*p* = 0.008, Cramer’s V: 0.230), primarily reported as first-perceived beneficial effects by very satisfied patients. Discrepancies in the most relevant perceived effects were rated by participants with different levels of satisfaction, particularly regarding the decrease in suicidal thoughts (*p* = 0.003, Cramer’s V: 0.255) and the recovery of physical energy (*p* < 0.001, Cramer’s V: 0.289). As for quality of life, partially satisfied, satisfied, and very satisfied patients reported, respectively, a mild (74.4%), moderate (49.5%), and significant (93.2%) impact of ESK-NS, while the unsatisfied group declared no impact (87%; *p* < 0.001, Cramer’s V: 0.669). The most improved areas of functioning in the “partially satisfied”, “satisfied”, and “very satisfied” groups were the affective–relational (55.8%, 48%, 66.1%) and social (41.9%, 46%, 66.1%) ones, with Cramer’s V effect sizes, respectively, of 0.294 and 0.276. Only very satisfied patients described significant improvements in academic–occupational functioning (64.4%, *p* < 0.001, Cramer’s V: 0.422), whereas unsatisfied participants stated no change in any area of functioning during or after treatment in 65.2% of cases (*p* < 0.001, Cramer’s V: 0.412). The most uncomfortable side effects (i.e., motor retardation/sedation, dizziness, nausea, vomiting, dissociative experiences/symptoms, and increased blood pressure) or their absence were comparable across groups (all *p* > 0.05), as well as number of hospitalizations in psychiatric settings due to a depressive recurrence after the end of treatment (*p* = 0.17). All information about the effects of ESK-NS as perceived by the overall sample and by the four subgroups is included in Table 2.

Post hoc analyses clarified the specific differences between groups. Specifically, in terms of first-perceived beneficial effects, very satisfied patients reported significantly greater improvement in depressed mood (vs. unsatisfied *p* < 0.001, vs. satisfied *p* = 0.003) and reduction in restlessness/sluggishness (vs. unsatisfied *p* = 0.001), feelings of guilt (vs. partially satisfied *p* = 0.002), and ideas of death/thoughts of suicide (vs. partially satisfied and satisfied, both *p* < 0.001). As for the most significant benefits, very satisfied patients experienced greater recovery of physical energy (vs. partially satisfied *p* < 0.001) and reduction in ideas of death/thoughts of suicide (vs. partially satisfied *p* = 0.002). Regarding quality of life, they scored significantly higher than all other groups (*p* < 0.001). In functional areas, as well, very satisfied participants showed greater improvements than the unsatisfied group across all domains (all *p* < 0.001), significantly surpassing partially satisfied (*p* < 0.001) and satisfied (*p* < 0.001) participants in the academic–occupational one.

**Figure 1:**
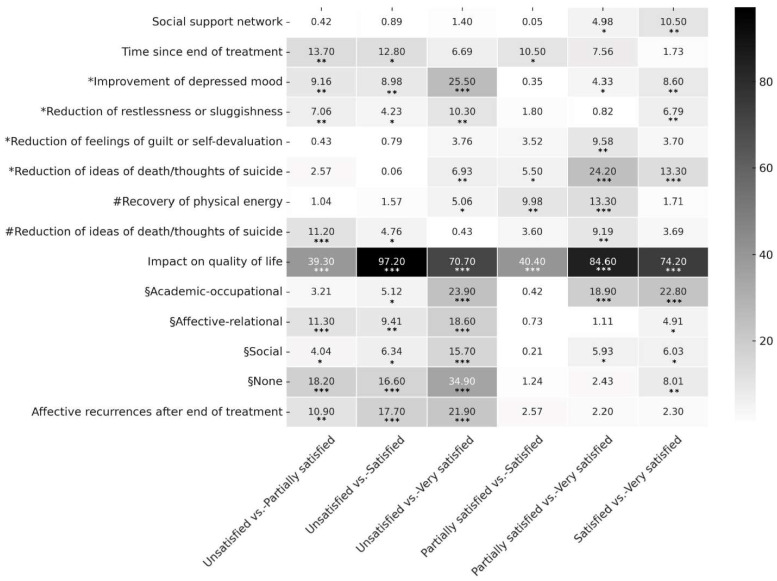
Heatmap of pairwise comparisons between subgroups for the variables of interest. * First perceived beneficial effects; # most significant beneficial effects; § areas of functioning. Shades of gray indicate the magnitude of differences between groups, with darker tones representing greater differences and higher statistics. Numeric values represent statistics from Chi-square/Fisher’s exact tests, with * *p* < 0.05, ** *p* < 0,01, *** *p* < 0.001 after Bonferroni correction.

Only a quarter of the overall sample (N = 57, 24.1%) described affective recurrences after treatment discontinuation (*p* < 0.001, Cramer’s V: 0.250), with unsatisfied participants showing higher rates compared to partially satisfied (*p* = 0.004), satisfied (*p* < 0.001), and very satisfied (*p* < 0.001) ones. All results of post hoc comparisons are illustrated in Figure 1 and summarized in Supplementary Material S2.

The most frequent suggestions proposed by participants for improving the experience were an increased frequency of ESK-NS administration (60.9%) and the possibility of at-home administration (44.2%), followed by the provision of psychological support during the observation period (19.1%). About half of the sample (44%) reported no need for further improvements.

### AI-Assisted Thematic Analysis

**Figure 2:**
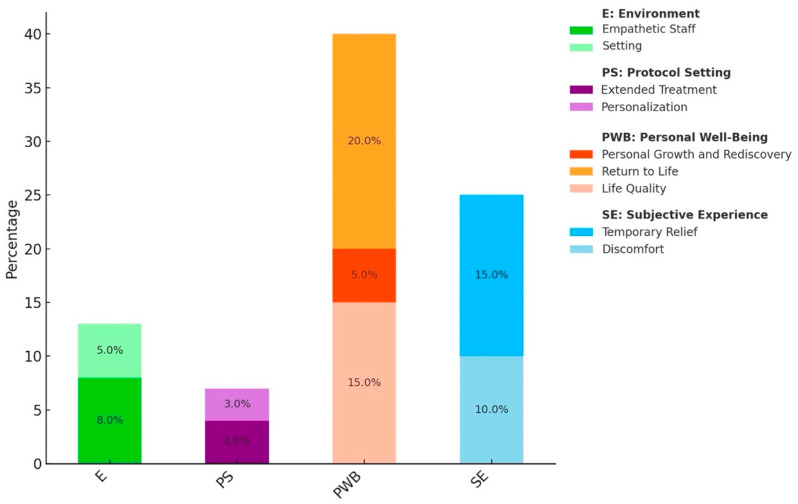
Recurring topics extracted from responses to the open-ended question and categorized through OpenAI.

The AI-assisted qualitative analysis identified recurring themes within the responses to the open-ended question. The primary themes were extracted and categorized in four primary domains, according to their content: personal well-being, subjective experience, environment, and protocol setting. Subcategories emerged within each theme, further detailing the spectrum of patient experiences, and were refined based on expert review and validated against the graphical representation of findings. Specifically, personal well-being includes the following topics: a sense of reconnecting with the inner self, rediscovering what is meaningful, and setting new personal goals, which is described as transformative and healing (i.e., Personal Growth and Rediscovery); the ability to participate in daily life activities, previously lost, with a renewed sense of purpose (i.e., Return to Life); and enhancement of energy levels and day-to-day quality of life, even without a full symptom remission (i.e., Life Quality). Subjective experience summarizes the following issues: relief moments and emotional enhancement perceived during and immediately after the administration, though these effects often diminish over time for many patients (i.e., Temporary Relief); potential influence of side effects on the experience and psychological distress deriving from dissociation if proper guidance on managing these sensations is missing (i.e., Discomfort). As for the environment, the presence of a supportive team with empathetic, competent doctors and nurses appears to be pivotal and appreciated by participants (i.e., Empathetic Staff), as well as the setting itself, with a desire for more comfort or the possibility to listen to neutral music to promote relaxation and emotional stability during observation phases (i.e., Setting). Last, as for the protocol setting, many patients express a wish for a longer duration or increased frequency of treatment sessions (i.e., Extended Treatment) and suggest considering greater flexibility and more tailored plans to suit individual needs (i.e., Personalization). A summary of the distribution of thematic clusters is illustrated in Figure 2.

## 4. Discussion

To our knowledge, the REAL-ESKperience study is the first to adopt a national, multicentric approach to explore the perspectives of a real-world sample of patients with TRD regarding the experience with intranasal esketamine through a dedicated, self-report survey. By combining structured and open-ended items, the study provides meaningful insights for a novel patient-centered understanding of treatment outcomes that extends beyond traditional symptomatic assessments. Our findings suggest that patients perceive the effects of ESK-NS as not limited to mood improvement, with a positive impact on quality of life, functionality, and personal well-being.

A substantial proportion of participants reported positive effects on depressive symptoms within the first three months of treatment, in line with a greater response to ESK-NS at three months described in real-world settings [13]. In our sample, the majority of patients declared a certain degree of satisfaction with treatment experience and a positive impact on quality of life and on levels of functioning, with the affective–relational and social areas being the most improved across satisfied participants. As for the symptomatic dimension, the reduction in depressed mood and suicidal thoughts was perceived among the first beneficial effects and considered as the most meaningful, particularly by the most satisfied participants.

Previous results from patients’ self-reports suggest that esketamine induced the most desired changes typically sought during antidepressant treatment, although there was variability in the onset of improvement across different symptomatic domains [24]. Indeed, patients’ experiences with intranasal esketamine have been less investigated compared to those with intravenous ketamine, and it has been described that subjective effects may vary between and within individuals [22] as well as compared to clinicians’ assessments, especially in specific domains like anhedonia and suicidal thoughts [19]. Depressive disorder with suicidal ideation represents a particularly severe subtype with more intense symptoms [30] and a worse treatment response compared to depression without suicidal ideation [31]. Although high-baseline suicidality has been associated with a reduced likelihood of response to ESK-NS at one and three months of treatment [14], glutamatergic antagonists, including intravenous ketamine and ESK-NS, have been shown to reduce suicide ideation and prevent suicidal behavior in TRD [20,32,33,34,35]. Indeed, ketamine and esketamine are known to exert favorable effects on neurobiological mechanisms involved in suicide pathogenesis independently of the underlying psychiatric illness [19,36,37,38]. These results regarding the perceived effectiveness of ESK-NS in reducing suicidal thoughts also seem to align with its approval as a short-term acute treatment for the rapid alleviation of depressive symptoms that clinicians classify as a psychiatric emergency, potentially including suicidality [39].

Interestingly, patients from the unsatisfied group perceived a reduction in depressed mood (39.1%) and suicidal thoughts (30.4%) as the first beneficial changes and considered both (33.3%) among the most significant effects of ESK-NS, although without improved life-quality and overall functioning. Such limited improvement can indicate that the therapeutic effects of esketamine may not universally translate into functional gains despite symptoms reduction, suggesting the need for additional support or adjunctive interventions for some patients [40]. Further, unsatisfied participants reported having experienced more affective recurrences since treatment-discontinuation than participants belonging to the other groups. It is known that the relapsing–remitting course of, M.D.D.; with a mean of up to four depressive episodes per patient, is associated with an increasing severity alongside the number of recurrences [41,42]. Given that the unsatisfied group also had the highest prevalence of patients who had discontinued ESK-NS for more than one year and the lowest proportion of participants still on treatment at the time of the survey completion, it could be argued that the interplay of all these factors may have influenced their ratings of the experience [43].

Beyond symptomatic improvements, many participants described broader subjective experiences like increased energy, reconnection with life activities, and personal growth, illustrating esketamine’s potential impact on psychosocial and personal well-being. These themes, identified through AI-assisted analysis, mirror findings from prior qualitative studies on esketamine reporting improvements in self-efficacy and interpersonal relationships as well as restored functionality and day-to-day life after treatment [22,24].

The absence of uncomfortable side effects was reported by 30% of the sample. These results are consistent with studies also supporting the good safety and tolerability profile for ESK-NS in the long-term [13,24,44] and might help reduce some clinicians’ and patients’ concerns about the extent of post-administration dissociation [45]. Nevertheless, many participants reported moments of psychological distress related to dissociative effects during the administration, in line with experiences of detachment commonly described in the literature [46,47,48,49]. Continued support should be provided throughout all stages of treatment, and some interventions have been proposed to mitigate and convert these transient experiences into therapeutic advantages [50]. In addition, detachment experiences may be coupled with themes of openness and spaciousness that can induce a transient cessation of negative thoughts and feelings. So far, the following strategies have been suggested: adequate education with the instruction of calming techniques; preparation for the post-administration states and expectation management; psychological and emotional support; and setting optimization for patient comfort (e.g., use of music), and presence of empathetic, caring staff [22]. These suggestions align with themes that emerged from this study’s AI-assisted analysis, leaving open the question of whether combining and delivering at the optimal timing psychotherapies and the above interventions could enhance the benefits of esketamine [40].

To our knowledge, only another study has characterized self-reported changes in adult patients with TRD treated with ESK-NS, focusing on emotional health, daily functioning, and treatment satisfaction. However, the small sample size and the inclusion of only patients still on treatment and responding to ESK-NS may limit the generalizability of results [24]. Beyond the emphasis on patients’ perspectives, the multicentric design, and large sample size, which are the main strengths of our study, there are several limitations that should be acknowledged. First, the evaluation of satisfaction and perceived improvement was based on self-reported measures and may be associated with recall bias. Detailed clinical data, including a family history of psychiatric diseases, are missing and limit the possibility of correlating subjective satisfaction with objective outcomes. Generalizability may also be constrained by regional variability in healthcare access and by the lack of a control group, which prevents direct comparison with alternative treatments. Moreover, the cross-sectional design and the variability in treatment duration, ranging from three to over nine months, do not allow for conclusions on long-term satisfaction, underscoring the need for longitudinal studies with extended follow-up, even considering the suggestion of extended treatment among patients’ proposed improvements. Additionally, this study did not involve experts by experience in the design or analysis phases, which may have limited the depth and contextual sensitivity of the data interpretation. Finally, while the use of AI for thematic analysis enhanced efficiency and organization, it poses challenges in terms of reproducibility and nuanced interpretation. Standardized prompts and manual review were used to improve reliability. However, the evolving nature of large language models requires future studies where AI-assisted text analysis is combined with traditional qualitative methods to enhance consistency [51]. Indeed, human oversight remains essential to ensure that AI-generated themes accurately describe patient experiences, reinforcing the need for rigorous validation and best practices in AI-driven qualitative research [52].

## 5. Conclusions

The burden of illness in depression is multidimensional and encompasses not only symptom severity but also impaired functioning, reduced quality of life, and dissatisfaction with health, occupation, and social activities [43]. While traditional models of depression primarily focus on neurobiological dysfunction and symptomatology, person-centered approaches offer a broader perspective by considering patients holistically and beyond clinical diagnoses [53]. Similar approaches recognize that psychological well-being is a multifaceted construct, incorporating various components such as the search for meaning and hope, and promote a more comprehensive understanding of recovery that transcends symptom remission. This perspective challenges the conceptualization of health as the mere absence of illness and paves the way for more individualized recovery trajectories [54,55].

Within this background, the REAL-ESKperience study highlights the clinical and subjective benefits of ESK-NS, demonstrating significant self-perceived improvements in depressive symptoms, suicidal thoughts, and overall quality of life in patients with TRD. By capturing patient perspectives, this study explores the multidimensional impact of esketamine, particularly the effects on functionality and personal growth, highlighting how much it extends beyond symptom reduction. These findings align with recent evidence suggesting that ESK-NS contributes to functional recovery and enhances workplace productivity compared to other augmentation strategies commonly used in clinical practice for managing TRD [56]. Notably, our results support this perspective, showing that participants who reported higher satisfaction with ESK-NS also perceived greater functional improvements, particularly in the academic–occupational domain.

However, the variability in patient satisfaction suggests that further personalization of treatment might be useful to fully optimize the benefits of ESK-NS for diverse patient profiles. Despite the growing emphasis on treatment personalization, structured guidelines for tailoring ESK-NS protocols based on individual patient characteristics are still lacking. Future research with longitudinal and controlled studies is needed to confirm these findings and to evaluate the sustained nature of subjective improvements. Additionally, while this study captures the perception of esketamine’s effects over varying treatment durations, the persistence of self-perceived benefits and their evolution over a longer time frame should be further explored to optimize long-term care for patients with TRD. Integrating patient-centered approaches in future trials will be pivotal in informing clinical decisions, enhancing satisfaction with treatment, and improving therapeutic outcomes. Such efforts would possibly allow for a more comprehensive understanding of esketamine’s effectiveness from both subjective and objective perspectives.

## Figures and Tables

**Table 1:** Sociodemographic and clinical differences of sample (overall and groups with distinct levels of satisfaction).

Characteristics (n, %; M ± SD)	Total	Unsatisfied	Partially Satisfied	Satisfied	Very Satisfied	χ^2^/F	*p*
**Overall**	236 (100)	24 (10.2)	45 (19.1)	105 (44.4)	62 (26.3)		
*Sociodemographic features*							
**Age**	54.1 ± 14.1	58.8 ± 10.1	53 ± 15	53.4 ± 13.9	54.6 ± 14.7	1.69	0.176
**Gender**						4.34	0.227
Male	106 (44.9)	16 (65.2)	20 (44.2)	44 (42)	26 (42.4)		
Female	130 (55.1)	8 (34.8)	25 (55.8)	61 (58)	36 (57.6)		
**Education**						14.3	0.112
Primary	18 (7.5)	0 (0)	8 (17.9)	7 (6.3)	3 (5.2)		
Junior High	49 (20.6)	9 (38.1)	9 (20.5)	19 (17.7)	12 (19)		
High	108 (45.8)	9 (38.1)	21 (46.2)	50 (47.9)	28 (44.8)		
University	61 (26.1)	6 (23.8)	7 (15.4)	29 (28.1)	19 (31)		
**Employment** (yes)	112 (47.7)	17 (71.4)	17 (38.5)	48 (45.8)	30 (48.3)	6.22	0.102
**Marital status**						2.58	0.461
Without Partner	118 (50)	12 (47.6)	20 (43.6)	50 (47.9)	36 (58.6)		
With Partner	118 (50)	12 (52.4)	25 (56.4)	55 (52.1)	26 (41.4)		
**Social support network** (yes)	193 (81.8)	19 (81)	39 (87.2)	93 (88.5)	42 (67.2)	11.9	**0.008**
*Clinical characteristics*							
**Time since end of treatment**						27.5	**0.007**
Not applicable *	133 (56.6)	8 (33.3)	21 (46.2)	68 (64.5)	36 (58.6)		
1–3 months	24 (10.3)	1 (4.8)	10 (23.1)	8 (7.3)	5 (8.6)		
4–6 months	27 (11.2)	3 (9.6)	7 (15.4)	12 (11.5)	5 (8.6)		
7–12 months	25 (10.7)	4 (19)	6 (12.8)	7 (7.3)	8 (12.1)		
>12 months	27 (11.2)	8 (33.3)	1 (2.5)	10 (9.4)	8 (12.1)		
**Treatment Duration**						13.1	0.158
3 months	52 (22.1)	8 (35)	15 (33.3)	23 (21.9)	6 (10.4)		
4–6 months	55 (23)	4 (15)	13 (28.2)	24 (22.9)	14 (22.4)		
7–9 months	50 (21.1)	6 (25)	7 (15.4)	23 (21.9)	14 (22.4)		
>9 months	79 (33.8)	6 (25)	10 (23.1)	35 (33.3)	28 (44.8)		
**Psychological Support/Psychotherapy** (yes)	123 (52.3)	9 (38.1)	24 (53.8)	58 (55.2)	32 (51.7)	2.07	0.558

Abbreviations: M, mean; *p*, statistical significance; SD, standard deviation; * not applicable since treatment was still ongoing.

**Table 2:** Self-perceived effects of treatment with esketamine nasal spray.

Characteristics (n, %; M ± SD)	Total	Unsatisfied	Partially Satisfied	Satisfied	Very Satisfied	χ^2^/F	*p*
**Overall**	236 (100)	24 (10.2)	45 (19.1)	105 (44.4)	62 (26.3)		
**Latency of beneficial effects**						25.3	0.05
Immediately after the first administrations	27 (11.2)	6 (23.8)	0 (0)	11 (11)	10 (15.3)		
Within the first two weeks	34 (14.3)	2 (9.5)	8 (18.6)	14 (13)	10 (15.3)		
Between the 2nd and 4th week	35 (14.8)	5 (19)	7 (16.3)	16 (15)	7 (11.9)		
Between the 1st and 2nd month	41 (17.9)	1 (4.8)	8 (18.6)	24 (23)	8 (13.6)		
Between the 2nd and 3rd month	40 (16.6)	1 (4.8)	15 (30.2)	13 (12)	11 (18.6)		
After the 3rd month	59 (25.2)	9 (38.1)	7 (16.3)	27 (26)	16 (25.3)		
**First-perceived beneficial effects**							
Improvement in depressed mood	176 (74.7)	9 (39.1)	34 (76.7)	76 (72)	57 (91.5)	24.7	**<0.001**
Increased ability to experience pleasure/interest	80 (33.8)	3 (13)	14 (30.2)	36 (34)	27 (44.1)	7.46	0.06
Regularization of appetite/body weight	39 (16.4)	3 (13)	4 (9.3)	20 (19)	12 (18.6)	2.47	0.48
Regularization of sleep–wake rhythm	57 (24.4)	3 (13)	8 (18.6)	27 (26)	19 (30.5)	3.72	0.29
Reduction in restlessness or sluggishness	49 (20.9)	0 (0)	11 (25.6)	17 (16)	21 (33.9)	14.1	**0.003**
Recovery of physical energy	81 (34.2)	4 (17.4)	21 (46.5)	34 (32)	22 (35.6)	6.05	0.11
Reduction in feelings of guilt or self-devaluation	39 (16.4)	2 (8.7)	2 (4.7)	17 (16)	18 (28.8)	11.9	**0.008**
Improvement in cognitive functioning/reduction in indecision	71 (30.2)	3 (13)	12 (27.9)	35 (33)	21 (33.9)	4.07	0.25
Reduction in ideas of death/thoughts of suicide	87 (36.9)	7 (30.4)	6 (14)	35 (33)	39 (62.7)	27.7	**<0.001**
Reduction in anxiety symptoms	73 (30.7)	2 (8.7)	15 (32.6)	34 (32)	22 (35.6)	6.05	0.11
**Most significant beneficial effect**							
Improvement in depressed mood	120 (50.9)	8 (33.3)	27 (59)	51 (49)	34 (55.2)	4.18	0.24
Increased ability to experience pleasure/interest	24 (10.3)	1 (4.8)	5 (10.3)	12 (11.5)	6 (10.3)	0.838	0.84
Regularization of appetite/body weight	4 (1.9)	1 (4.8)	0 (0)	2 (2.1)	1 (1.7)	1.73	0.63
Regularization of sleep–wake rhythm	3 (1.4)	0 (0)	1 (2.6)	1 (1)	1 (1.7)	0.813	0.85
Reduction in restlessness or sluggishness	8 (3.3)	0 (0)	1 (2.6)	6 (5.2)	1 (1.7)	2.35	0.51
Recovery of physical energy	22 (9.3)	3 (14.3)	11 (25.6)	7 (6.3)	1 (1.7)	17.9	**<0.001**
Reduction in feelings of guilt or self-devaluation	5 (2.3)	0 (0)	0 (0)	3 (3.1)	2 (3.4)	2.01	0.57
Improvement in cognitive functioning/reduction in indecision	18 (7.9)	2 (9.5)	3 (7.7)	10 (9.4)	3 (5.2)	0.953	0.81
Reduction in ideas of death/thoughts of suicide	39 (16.8)	8 (33.3)	1 (2.6)	14 (13.5)	16 (25.9)	13.9	**0.003**
Reduction in anxiety symptoms	28 (12.1)	2 (9.5)	10 (23.1)	11 (10.4)	5 (8.6)	5.45	0.14
**Impact on quality of life**						300	**<0.001**
None	27 (11.6)	21 (87)	4 (9.3)	1 (1)	1 (1.7)		
Mild	65 (27.2)	2 (8.7)	34 (74.4)	28 (26.3)	1 (1.7)		
Moderate	61 (25.9)	1 (4.3)	6 (14)	52 (49.5)	2 (3.4)		
Significant	83 (35.3)	0 (0)	1 (2.3)	24 (23.2)	58 (93.2)		
**Most improved areas of functioning**							
Academic–occupational	77 (32.9)	1 (4.3)	9 (20.9)	27 (26)	40 (64.4)	40	**<0.001**
Affective–relational	119 (50.7)	3 (13)	25 (55.8)	50 (48)	41 (66.1)	19.4	**<0.001**
Social	112 (47.6)	4 (17.4)	19 (41.9)	48 (46)	41 (66.1)	17.2	**<0.001**
None	48 (20.4)	16 (65.2)	6 (14)	23 (22)	3 (5.1)	38.2	**<0.001**
**Most uncomfortable side effect**							
Motor retardation/sedation	94 (39.6)	6 (26.1)	20 (44.2)	37 (35)	31 (49.2)	5.27	0.15
Dizziness	93 (39.5)	6 (26.1)	20 (44.2)	36 (35)	31 (49.2)	5.27	0.15
Nausea	38 (16.4)	3 (13)	7 (16.3)	20 (19)	8 (13.6)	1.03	0.79
Vomiting	20 (8.9)	1 (4.3)	7 (16.3)	9 (9)	3 (5.1)	4.54	0.21
Dissociative experiences/symptoms	60 (25.3)	5 (21.7)	12 (25.6)	24 (23)	19 (30.5)	1.28	0.73
Increased blood pressure	20 (8.4)	2 (8.7)	6 (14)	9 (8)	3 (5.1)	2.58	0.46
None	71 (30.2)	10 (43.5)	9 (20.9)	34 (32)	18 (28.8)	3.88	0.27
**Affective recurrences after end of treatment**						28	**<0.001**
Not applicable *	133 (56.7)	6 (26.1)	22 (50)	66 (63)	39 (62.7)		
Yes	57 (24.1)	16 (65.2)	11 (23.8)	22 (21)	8 (13.6)		
No	46 (19.2)	2 (8.7)	12 (26.2)	17 (16)	15 (23.7)		
**Hospitalizations ^#^ after end of treatment**						9.05	0.17
Not applicable *	117 (49.8)	8 (33.3)	21 (46.2)	62 (59.4)	26 (42.1)		
Yes	6 (2.8)	1 (4.8)	0 (0)	3 (3.1)	2 (3.5)		
No	113 (47.4)	15 (61.9)	24 (53.8)	40 (37.5)	34 (54.4)		

Abbreviations: M, mean; *p*, statistical significance; SD, standard deviation; * not applicable since treatment was still ongoing; ^#^ hospitalizations in psychiatric settings due to a depressive recurrence.

## Data Availability

The data are not publicly available due to data protections regulations.

## Supplementary Materials

The following supporting information can be downloaded at https://www.mdpi.com/article/10.3390/jpm15040161/s1. File S1. Survey: “Your Experience with Esketamine Nasal Spray”; File S2: Pairwise comparisons between subgroups with post-hoc correction (significant results after Bonferroni correction in bold).

## Appendix A

REAL-ESKperience Study Group: Bardi F., Calderoni C., and Giannico A. M.: Department of Neuroscience, Section of Psychiatry, Università Cattolica del Sacro Cuore, Rome; Baglioni A.: Department of Clinical and Experimental Sciences, University of Brescia, Brescia, Italy; Galluzzo A.: Department of Mental Health and Addiction Services, ASST Spedali Civili of Brescia, Brescia, Italy; Pinna M.: Unit of Clinical Psychiatry, University Hospital Agency of Cagliari, Cagliari, Italy; Barone L. and Conti D.: Department of Clinical and Experimental Medicine, Institute of Psychiatry, University of Catania, Catania, Italy; Giacomini G.: Department of Neuroscience, Rehabilitation, Ophthalmology, Genetics, Maternal and Child Health (DINOGMI), Section of Psychiatry, University of Genova, Genova, Italy; Cornaggia R. D. and Casetti V.: Department of Mental Health, Fondazione IRCCS San Gerardo dei Tintori, Monza, Italy; Raffone F.: Department of Mental Health, ASL Napoli 1 Centro, Napoli, Italy; Di Natale C.: Department of Mental Health, ASL 4, Teramo, Italy; Di Salvo G. and Garrone C.: San Luigi Gonzaga University Hospital, Department of Neurosciences “Rita Levi Montalcini”, Università di Torino, Torino, Italy; Garofalo S. and Grilli G.: Chair of Psychiatry, Department of Systems Medicine, Tor Vergata, Rome, Italy.
