# Supplementary material for: Patient Experience with Intranasal Esketamine in Treatment-Resistant Depression: Insights from a Multicentric Italian Study (REAL-ESKperience)
Source: J Pers Med. 2025 Apr 21;15(4):161. doi: 10.3390/jpm15040161 (PMC12029048; doi:10.3390/jpm15040161)
Supplement: Supplementary file 1 [file jpm-15-00161-s001.zip › S1_Survey.pdf]

Journal of Personalized Medicine

Patient Experience with Intranasal Esketamine in Treatment-Resistant Depression: Insights from a Multicentric Italian Study (REAL-ESKperience)

Di Nicola M., Pepe M.*, d'Andrea G., Marcelli I., Pettorruso M., Andriola I., Barlati S., Carminati M., Cattaneo C. I., Clerici M., De Berardis D., De Filippis S., Dell'Osso B. M., Di Lorenzo G., Maina G., Manchia M., Marcatili M., Martiadis V., Niolu C., Petralia A., Rosso G., Serafini G., Signorelli M. S., Vannucchi T., Vismara M., Zanardi R., Vita A., Sani G., Martinotti G, REAL-ESKperience Study Group

[REAL-ESKperience Study Group: Bardi F., Calderoni C., Giannico A. M., Baglioni A., Galluzzo A., Pinna M., Barone L., Conti D., Giacomini G., Cornaggia R. D., Casetti V., Raffone F., Di Natale C., Di Salvo G., Garrone C., Garofalo S., Grilli G.]

* Corresponding author:
Maria Pepe, MD
Department of Neuroscience, Section of Psychiatry, Università Cattolica del Sacro Cuore, Rome, Italy
Department of Psychiatry, Fondazione Policlinico Universitario Agostino Gemelli IRCCS, Rome, Italy
Largo Francesco Vito 1, Rome 00168, Italy
Tel: +39 06 30154122
E-mail: maria.pepe@unicatt.it

Survey. "*Your Experience with Esketamine Nasal Spray*"

This questionnaire aims to evaluate your experience with Esketamine nasal spray from your perspective. Completion of this survey will require only a few minutes.
Please be assured that all data collection complies fully with applicable privacy regulations.

1. **Year of birth: __________**

2. **Gender:**
   a) Male
   b) Female
   c) Other

3. **Education:**
   a) Primary school
   b) Junior high school
   c) High school
   d) University degree or higher

4. **Employment:**
   a) No
   b) Yes

5. **Marital status:**
   a) Single
   b) Cohabiting/married
   c) Separated/divorced
   d) Widower

6. **Do you have a family or social network support?**
   a) No
   b) Yes

7. **How long has it been since you discontinued treatment with Esketamine?**
   a) Not applicable (treatment still ongoing)
   b) 1-3 months

c) 4-6 months
d) 7-12 months
e) More than 12 months

8. For how many months did you receive Esketamine treatment?
a) 3 months
b) 4-6 months
c) 7-9 months
d) More than 9 months

9. During treatment with Esketamine, did you receive psychological support or undergo psychotherapy?
a) No
b) Yes

10. How soon after initiating Esketamine treatment did you first notice beneficial effects?
Please select only one response.
a) Immediately after the first administrations
b) Within the first two weeks
c) Between the second and fourth week
d) Between the first and second month
e) Between the second and third month
f) After the third month

11. What beneficial effects did you perceive first?
You may select more than one response.
a) Improvement of depressed mood
b) Increased ability to experience pleasure and/or interest
c) Regulation of appetite and/or body weight
d) Regulation of sleep-wake rhythm
e) Reduction of restlessness or sluggishness
f) Recovery of physical energy
g) Reduction of feelings of guilt or self-devaluation
h) Improvement of cognitive functioning and/or reduction of indecision
i) Reduction of ideas of death and/or thoughts of suicide
j) Reduction of anxiety symptoms

12. **Among the above-mentioned beneficial effects, which was the most significant for you?**
Please select only one response.
a) Improvement of depressed mood
b) Increased ability to experience pleasure and/or interest
c) Regulation of appetite and/or body weight
d) Regulation of sleep-wake rhythm
e) Reduction of restlessness or sluggishness
f) Recovery of physical energy
g) Reduction of feelings of guilt or self-evaluation
h) Improvement of cognitive functioning and/or reduction of indecision
i) Reduction of ideas of death and/or thoughts of suicide
j) Reduction of anxiety symptoms

13. If you experienced any side effects during Esketamine treatment, which were the most uncomfortable?
You may select more than one response.
a) Motor retardation or sedation
b) Dizziness
c) Nausea
d) Vomiting
e) Dissociative experiences or symptoms
f) Increase in blood pressure
g) None

14. Which areas of functioning demonstrated the greatest improvement as a result of Esketamine treatment?
You may select more than one response.
a) School/work (e.g., resumption of activities/improved performance in studies or work)

b) Affective-relational (e.g., recovery or improvement of relationships with partner/family/friends)
c) Social (e.g., increased interest or pleasure in social interactions/more active participation in recreational activities)
d) None of the above

15. What was the impact of Esketamine treatment on your quality of life?
a) None
b) Mild
c) Moderate
d) Significant

16. Following the discontinuation of treatment, did you experience a recurrence of significant depressive symptoms?
a) Not applicable (treatment still ongoing)
b) Yes, within 3 months
c) Yes, between 3 and 6 months
d) Yes, between 6 and 12 months
e) Yes, after one year
f) No

17. After treatment, did you undergo any hospitalizations due to acute and/or severe depressive states?
a) Not applicable (treatment still ongoing)
b) Yes, within 3 months
c) Yes, between 3 and 6 months
d) Yes, between 6 and 12 months
e) Yes, after one year
f) No

18. Overall, how would you rate your experience with Esketamine treatment?
a) Unsatisfying
b) Partially satisfying
c) Satisfying
d) Very satisfying

19. What modifications would you recommend to enhance your experience with Esketamine treatment?
You may select more than one response.
a) Increased frequency of administrations
b) Lower frequency of administrations
c) Longer observation period
d) Shorter observation period
e) Presence of other patients (e.g., I would have preferred to receive treatment with others)
f) Absence of other patients (e.g., I would have preferred to receive treatment alone)
g) Presence of family members/care-givers (e.g., I would have preferred to receive treatment with their support)
h) Absence of family members/care-givers (e.g., I would have preferred to receive treatment without their support)
i) Association of psychotherapy during the observation period
j) At-home administrations
k) Nothing to change

20. Please describe your experience with Esketamine in a few words:
____

____
