# Supplementary material for: Patient Experience with Intranasal Esketamine in Treatment-Resistant Depression: Insights from a Multicentric Italian Study (REAL-ESKperience)
Source: J Pers Med. 2025 Apr 21;15(4):161. doi: 10.3390/jpm15040161 (PMC12029048; doi:10.3390/jpm15040161)
Supplement: Supplementary file 1 [file jpm-15-00161-s001.zip › S2_Supplementary Table.pdf]

Supplementary Table. Pairwise comparisons between subgroups with post-hoc correction.

	Variable	**Unsatisfied** *vs.* **Partially satisfied**		**Unsatisfied** *vs.* **Satisfied**		**Unsatisfied** *vs.* **Very satisfied**		**Partially satisfied** *vs.* **Satisfied**		**Partially satisfied** *vs.* **Very satisfied**		**Satisfied** *vs.* **Very satisfied**	
		χ^2/F	*p*	χ^2/F	*p*	χ^2/F	*p*	χ^2/F	*p*	χ^2/F	*p*	χ^2/F	*p*
	**Social support network**	0.42	0.319	0.89	0.346	1.40	0.236	0.05	0.824	4.98	0.026	10.50	**0.001**
	**Time since end of treatment**	13.7	0.008	12.80	0.012	6.69	0.153	10.50	0.033	7.56	0.109	1.73	0.786
First-perceived beneficial effects	**Improvement of depressed mood**	9.16	**0.002**	8.98	**0.003**	25.50	**<.001**	0.35	0.556	4.33	0.037	8.60	**0.003**
	**Reduction of restlessness or sluggishness**	7.06	0.008	4.23	0.040	10.30	**0.001**	1.80	0.179	0.82	0.367	6.79	0.009
	**Reduction of feelings of guilt or self-devaluation**	0.43	0.512	0.79	0.372	3.76	0.052	3.52	0.061	9.58	**0.002**	3.70	0.054
	**Reduction of ideas of death/thoughts of suicide**	2.57	0.109	0.06	0.813	6.93	0.008	5.50	0.019	24.20	**<.001**	13.30	**<.001**
Most significant beneficial effect	**Recovery of physical energy**	1.04	0.309	1.57	0.211	5.06	0.024	9.98	**0.002**	13.30	**<.001**	1.71	0.191
	**Reduction of ideas of death/thoughts of suicide**	11.20	**<.001**	4.76	0.029	0.43	0.513	3.60	0.058	9.19	**0.002**	3.69	0.055
	**Impact on quality of life**	39.30	**<.001**	97.20	**<.001**	70.70	**<.001**	40.40	**<.001**	84.6	**<.001**	74.20	**<.001**
Most improved areas of functioning	**Academic-occupational**	3.21	0.073	5.12	0.024	23.90	**<.001**	0.42	0.518	18.9	**<.001**	22.80	**<.001**
	**Affective-relational**	11.30	**<.001**	9.41	**0.002**	18.60	**<.001**	0.73	0.391	1.11	0.291	4.91	0.027
	**Social**	4.04	0.045	6.34	0.012	15.70	**<.001**	0.21	0.648	5.93	0.015	6.03	0.014
	**None**	18.20	**<.001**	16.60	**<.001**	34.90	**<.001**	1.24	0.266	2.43	0.119	8.01	**0.005**
	**Affective recurrences after end of treatment**	10.90	**0.004**	17.70	**<.001**	21.90	**<.001**	2.57	0.277	2.20	0.333	2.30	0.316

Significant results after Bonferroni correction in **bold**.
